# Genome-Wide Analysis of Differentially Expressed miRNAs and Their Associated Regulatory Networks in Lenses Deficient for the Congenital Cataract-Linked Tudor Domain Containing Protein TDRD7

**DOI:** 10.3389/fcell.2021.615761

**Published:** 2021-02-16

**Authors:** Deepti Anand, Salma Al Saai, Sanjaya K. Shrestha, Carrie E. Barnum, Shinichiro Chuma, Salil A. Lachke

**Affiliations:** ^1^Department of Biological Sciences, University of Delaware, Newark, DE, United States; ^2^Center for Bioinformatics & Computational Biology, University of Delaware, Newark, DE, United States; ^3^Institute for Frontier Medical Sciences, Kyoto University, Kyoto, Japan

**Keywords:** cataract, lens aberration, microRNA, microarray, TDRD7, gene regulatory networks, eye development and function

## Abstract

Mutations/deficiency of *TDRD7*, encoding a tudor domain protein involved in post-transcriptional gene expression control, causes early onset cataract in humans. While Tdrd7 is implicated in the control of key lens mRNAs, the impact of *Tdrd7* deficiency on microRNAs (miRNAs) and how this contributes to transcriptome misexpression and to cataracts, is undefined. We address this critical knowledge-gap by investigating *Tdrd7*-targeted knockout (*Tdrd7-/-*) mice that exhibit fully penetrant juvenile cataracts. We performed Affymetrix miRNA 3.0 microarray analysis on *Tdrd7-/-* mouse lenses at postnatal day (P) 4, a stage preceding cataract formation. This analysis identifies 22 miRNAs [14 over-expressed (miR-15a, miR-19a, miR-138, miR-328, miR-339, miR-345, miR-378b, miR-384, miR-467a, miR-1224, miR-1935, miR-1946a, miR-3102, miR-3107), 8 reduced (let-7b, miR-34c, miR-298, miR-382, miR-409, miR-1198, miR-1947, miR-3092)] to be significantly misexpressed (fold-change ≥ ± 1.2, *p*-value < 0.05) in *Tdrd7-/-* lenses. To understand how these misexpressed miRNAs impact *Tdrd7-/-* cataract, we predicted their mRNA targets and examined their misexpression upon *Tdrd7*-deficiency by performing comparative transcriptomics analysis on P4 and P30 *Tdrd7-/-* lens. To prioritize these target mRNAs, we used various stringency filters (*e.g.*, fold-change in *Tdrd7-/-* lens, iSyTE-based lens-enriched expression) and identified 98 reduced and 89 elevated mRNA targets for overexpressed and reduced miRNAs, respectively, which were classified as “top-priority” “high-priority,” and “promising” candidates. For *Tdrd7-/-* lens overexpressed miRNAs, this approach identified 18 top-priority reduced target mRNAs: *Alad*, *Ankrd46*, *Ceacam10*, *Dgat2*, *Ednrb*, *H2-Eb1*, *Klhl22*, *Lin7a*, *Loxl1*, *Lpin1*, *Npc1*, *Olfm1*, *Ppm1e*, *Ppp1r1a*, *Rgs8*, *Shisa4*, *Snx22* and *Wnk2*. Majority of these targets were also altered in other gene-specific perturbation mouse models (*e.g., Brg1*, *E2f1/E2f2/E2f3*, *Foxe3*, *Hsf4*, *Klf4*, *Mafg*/*Mafk*, *Notch*) of lens defects/cataract, suggesting their importance to lens biology. Gene ontology (GO) provided further insight into their relevance to lens pathology. For example, the *Tdrd7*-deficient lens capsule defect may be explained by reduced mRNA targets (*e.g., Col4a3*, *Loxl1*, *Timp2*, *Timp3*) associated with “basement membrane”. GO analysis also identified new genes (*e.g., Casz1*, *Rasgrp1*) recently linked to lens biology/pathology. Together, these analyses define a new Tdrd7-downstream miRNA-mRNA network, in turn, uncovering several new mRNA targets and their associated pathways relevant to lens biology and offering molecular insights into the pathology of congenital cataract.

## Introduction

Perturbations in lens development results in congenital cataract in humans and animal models (Graw, 2009; Shiels and Hejtmancik, 2019). Studies over the past several decades have led to a detailed understanding of the key signaling and transcriptional regulatory mechanisms that orchestrate the genetic program of lens development (Donner et al., 2006; Lachke and Maas, 2010; Cvekl and Zhang, 2017). However, compared to signaling and transcription, the impact of post-transcriptional gene expression control to organogenesis, in general, and lens development, in particular, remains relatively understudied (Lachke and Maas, 2011; Blackinton and Keene, 2014; Dash et al., 2016; Cvekl and Zhang, 2017). Post-transcriptional control of gene expression is defined as the regulation of any of the different events from the processing of pre-mRNA to the degradation of mRNA (Singh et al., 2015). Indeed, non-coding RNAs such as microRNAs (miRNAs) as well as RNA-binding proteins (RBPs) are involved in various post-transcriptional regulatory processes, including control over translation or decay of mRNA (Pasquinelli, 2012; Manning and Cooper, 2017; Hentze et al., 2018; O’Brien et al., 2018). Thus far, very few RBPs and post-transcriptional regulatory factors, including miRNAs, have been functionally implicated in lens development and cataract (Lorén et al., 2009; Lachke et al., 2011; Choudhuri et al., 2013; Shaham et al., 2013; Wolf et al., 2013; Xie et al., 2014; Dash et al., 2015, 2020; Siddam et al., 2018; Aryal et al., 2020; Barnum et al., 2020; Nakazawa et al., 2020; Shao et al., 2020). This limited information highlights a substantial knowledge-gap in lens and cataract research because post-transcriptional control represents critical mechanisms that allow precise calibration, in terms of dosage and spatiotemporal pattern, of the cellular proteome. Thus, these regulatory mechanisms may be significant for controlling mRNA and protein abundance in lens fiber cells – a cell fate that faces added challenges to regulate these basic processes as they undergo nuclear degradation in terminal differentiation (Dash et al., 2016).

In embryogenesis, expression of Tdrd7 (Tudor domain containing 7) is highly enriched in lens fiber cells and is conserved between aves and mammals, suggesting its critical function in lens development (Lachke et al., 2011). Indeed, *TDRD7* mutations or deficiency results in congenital cataract in humans, mouse and chicken (Lachke et al., 2011; Tanaka et al., 2011; Chen et al., 2017; Tan et al., 2019). Furthermore, single nucleotide polymorphisms in *TDRD7* are also linked to age-related cataract (Zheng et al., 2014), making it among the select few genes associated with both early- and late-onset cataract (Shiels and Hejtmancik, 2019). Tdrd7 contains three tudor domains and three OST-HTH (Oskar-Tdrd7-Helix turn helix)/LOTUS motifs (Hosokawa et al., 2007; Anantharaman et al., 2010; Callebaut and Mornon, 2010; Tanaka et al., 2011). Tudor domains are considered to facilitate interaction with methylated arginine or lysine residues within other proteins (Chen et al., 2011; Pek et al., 2012; Gan et al., 2019). The OST-HTH/LOTUS domains in Drosophila protein *oskar*, predicted to bind RNA (Anantharaman et al., 2010; Callebaut and Mornon, 2010), has been shown to interact with a dead-box helicase (Jeske et al., 2015, 2017). While Tdrd-family proteins have been implicated in the control of small RNAs, previous studies have primarily focused on their association with piwi-interacting RNAs (piRNAs) in the context of spermatogenesis (Pek et al., 2012; Gan et al., 2019). Interestingly, while its mutation or deficiency is linked to azoospermia in human and mouse (Lachke et al., 2011; Tanaka et al., 2011; Tan et al., 2019), Tdrd7 has been shown to function in repression of Line-1 retrotransposons but is not found to be essential for production of piRNAs in mouse spermatogenesis (Tanaka et al., 2011). However, the effect of *Tdrd7* deletion on other classes of small non-coding RNAs such as miRNAs in the lens, and its impact on lens development and cataract formation has not been addressed.

Previously, we used *Tdrd7*-deficient mice–which exhibit fully penetrant cataracts and re-capitulate features of the human lens defects–to gain insight into Tdrd7’s role in lens development (Lachke et al., 2011; Barnum et al., 2020). We showed that removal of *Tdrd7* results in misexpression of several lens expressed mRNAs. We also demonstrated that Tdrd7 protein closely associates with specific mRNAs, for example *Hspb1* mRNA, which may enable it to directly control its abundance in the lens. Here, we sought to examine the impact of Tdrd7 deletion on global miRNA abundance in the lens. We performed microarray-based profiling of miRNAs in *Tdrd7-/-* mouse lens at postnatal day (P) 4, which precedes detectable lens defects and overt cataract formation. We identified misexpressed miRNAs and predicted their mRNA targets in the lens. We then performed comparative analysis with *Tdrd7-/-* lens RNA-seq and microarray datasets to identify inversely associated mRNA targets of the misexpressed miRNAs. This allowed us to derive a Tdrd7-downstream miRNA-mRNA network that led to the identification of new candidate genes with potential function in the lens. Importantly, iSyTE analysis showed that similar numbers of overexpressed and reduced mRNA targets in *Tdrd7-/-* lens were enriched in normal lens, suggesting that Tdrd7 functions in optimal control of a subset of lens-enriched mRNAs likely via regulating miRNAs. Finally, in addition to informing on *Tdrd7* deficiency, the global lens miRNA profile generated in this study provides independent support for expression of some of the abundant miRNAs in normal lens development that were described in previous studies, involving for example, *in situ* hybridization assays (Conte et al., 2010; Karali et al., 2010; Khan et al., 2016). These are: miR-184, miR-26a, let-7b, let-7c, miR-204 and miR-125b, among others. Together these data identify new high-priority Tdrd7-downstream miRNA and mRNA targets, thereby advancing our understanding of how this conserved Tudor family protein functions to fine-tune lens transcriptome in development and how its misregulation impacts cataract pathology.

## Materials and Methods

### Mouse Studies

Mice were maintained at the University of Delaware Animal Facility. Experimental protocols followed guidelines based on the Association for Research in Vision and Ophthalmology (ARVO) Statement for the use of animals in ophthalmic and vision research and were approved by the University of Delaware Institutional Animal Care and Use Committee (IACUC). The present studies were performed on *Tdrd7* targeted germline knockout (KO) mouse line (*Tdrd7^TM 1.1Chum^*, hereafter referred as *Tdrd7-/-*) that were genotyped as previously described (Tanaka et al., 2011).

### Sample Preparation and miRNA Microarray Analyses

For miRNA microarray, microdissected mouse lenses at postnatal day (P) 4 were collected from *Tdrd7-/-* and control (*Tdrd7*±, which does not develop cataract) in three biological replicates. Total RNA isolation was performed using the *mir*Vana^TM^ RNA isolation kit (Life Technologies, Grand Island, NY). Global expression profiling for miRNAs was performed using Affymetrix miRNA 3.0 arrays. Analysis of the raw expression datasets was performed under ‘R’ Statistical environment [http://www.r-project.org/index.html] using “Affy” packages. The datasets were background corrected, normalized and summarized using Robust Multi-array Average (RMA) method (Irizarry et al., 2003a, b). The obtained normalized miRNA expression values were subjected to downstream analysis using ‘limma’ package. Comparisons of control and *Tdrd7-/-* samples was carried out to identify highly and differentially expressed miRNAs with significant *p*-value (≥ 0.05) and absolute fold change (FC) ≥ ± 1.2. The detailed pipeline for Affymetrix microarray dataset analysis after data normalization is published elsewhere (Anand et al., 2015; Kakrana et al., 2018). The datasets generated in this study were deposited in GSE157061.

### qPCR Analysis of miRNAs in the Lens

Selected *Tdrd7-/-* lens misexpressed candidate miRNAs were analyzed by custom-designed PCR primers using miRCURY LNA miRNA PCR system (Qiagen, Germantown, MD). Total RNA was isolated from P4 *Tdrd7-/-* and control lenses in three biological replicates using miRNeasy mini kit (Qiagen Catalog: 217004). First-strand cDNA synthesis was performed using miRCURY LNA Universal RT kit (Qiagen Catalog: 339340) and miRNA expression was quantified using miRCURY LNA SYBR Green PCR kit (Qiagen Catalog: 339345) according to the manufacturer’s instructions. qPCR was run on BioRad CFX RT-PCR thermal cycler. MiRNA expression was normalized to miR-17, which exhibits robust expression in the lens and is not altered in *Tdrd7-/-* lens, as well as the housekeeping genes *Gapdh* and *Actb*. Relative expression was estimated using the 2^–ΔΔCT^ followed by statistical two-level nested analysis of variance test to calculate *p-*values.

### miRNA Target Prediction and Gene Ontology Enrichment Analysis of miRNA Targets

We performed miRNA target prediction and functional annotation analysis for differentially expressed miRNAs in *Tdrd7-/-* lenses. To retrieve predicted mRNA targets of miRNAs, the miRDB resource (Chen and Wang, 2020) that uses experimental data (*e.g.*, miRNA overexpression, publicly available CLIP-seq data) in their miRNA-mRNA target prediction algorithm, MirTarget, was used. The miRDB-identified mRNA targets with a probability score ≥ 50 were retrieved for further downstream analysis and were tested for their functional relevance to lens development using gene ontology (GO) analysis in DAVID bioinformatics tool (https://david.ncifcrf.gov). The top clusters with highest enrichment scores with functionally relevant GO categories at *p*-value ≤ 0.05 were presented. The TargetScan database (www.targetscan.org) was used to search for target mRNA conservation sites that match with the seed region of miRNAs across vertebrates (Lewis et al., 2005).

### Identification of Lens Enriched miRNA-mRNA Pairs

The miRNA target mRNAs were curated using lens gene-discovery bioinformatics tool-iSyTE (Lachke et al., 2012; Kakrana et al., 2018) to identify candidate miRNA-mRNA pairs (*i.e.*, miRNA and their predicted mRNA targets) associated with lens function and development. In this study, miRNA-mRNA pairs were tested for lens expression (expression ≥ 100) from P4 mRNA microarray dataset in iSyTE (Kakrana et al., 2018). To further assess candidate miRNA-mRNA pairs, we revisited the whole genome RNA-seq (GSE134384) and mRNA microarray datasets on *Tdrd7* null and control lenses at P4 (GSE25775) (Lachke et al., 2011) and performed an analysis to evaluated inverse correlation between differentially expressed ‘up’ and ‘down’ miRNA with their mRNA targets. The inverse correlation for elevated and reduced (used in the context of miRNAs) miRNA-mRNA pairs was tested using Pearson method implemented in R-package. Data was presented as miRNA-mRNA plots and miRNA-mRNA regulatory network was visualized as regulatory networks using cytoscape (https://cytoscape.org).

### Network Analysis of miRNA Target mRNAs

Candidate target mRNAs of the *Tdrd7-/-* lens differentially miRNAs were examined for differential expression by analyzing lens transcriptomics datasets in various gene-perturbation mouse models that develop lens defects/cataract. These include *Brg1* (dominant negative dnBrg1) at E15.5 (GSE22322), *E2f1:E2f2:E2f3* (triple lens-specific conditional knockout) at P0 (GSE16533), Foxe3 (transgenic mice with *Cryaa*-promoter based over-expression of *Foxe3* in lens fiber cells) at P2 (GSE9711), *Hsf4* (germline knockout) at P0 (GSE22362), *Klf4* (lens-specific conditional knockout) at E16.5 and P56 (GSE47694), *Mafg-/-:Mafk* ± (germline compound knockout) at P60 (GSE65500) and *Notch2* (lens-specific conditional knockout) at E19.5 (GSE31643). The network connectivity for mRNAs with these key lens factors were visualized using an open source tool Cytoscape (https://cytoscape.org).

## Results and Discussion

### Global miRNA Profiling of *Tdrd7-/-* Lens Prior to Formation of Cataract

**FIGURE 1:**
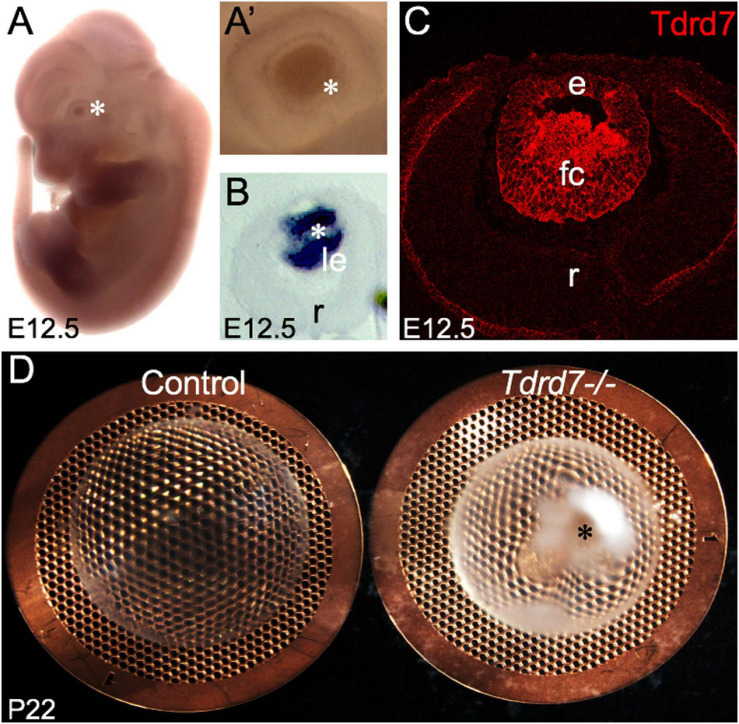
Tdrd7 is expressed in mouse embryonic lens development and its deficiency results in early onset cataract. **(A)** *In situ* hybridization of mouse embryonic day stage E12.5 with *Tdrd7*-specific probe shows Tdrd7 transcript signal in the eye lens (asterisk), **(A’)** also clearly observed at high-magnification (asterisk). **(B)** Section *in situ* hybridization confirms *Tdrd7* transcripts to be expressed in the mouse E12.5 lens (le) and demonstrates it to be primarily localized to fiber cells. **(C)** Immunofluorescence staining using antibody against Tdrd7 demonstrates its protein abundance (red) in the cytoplasm of lens fiber cells at E12.5. **(D)** *Tdrd7-/-* germline targeted knockout mouse lens show severe cataract (asterisk) compared to normal control at P22 stage. le, lens; r, retina; e, lens epithelium; fc, lens fiber cells.

**FIGURE 2:**
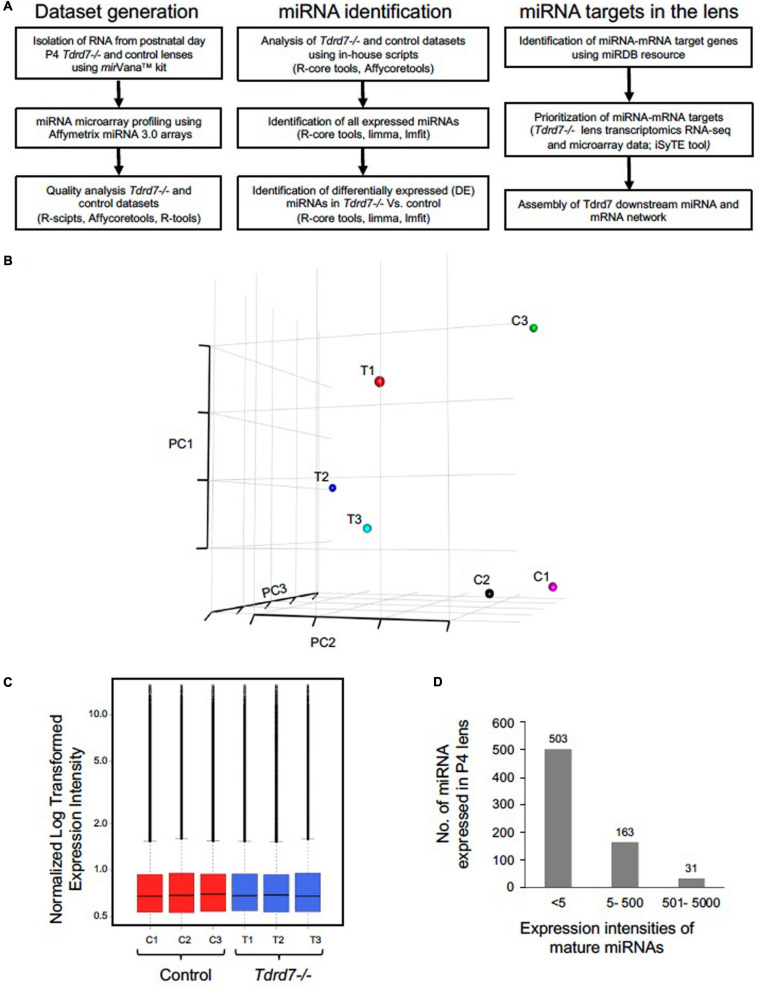
Microarray-based profiling of miRNAs in *Tdrd7-/-* lenses. **(A)** Step-by-step workflow showing bioinformatics tools and R-packages used for generation and analysis of post-natal day (P) 4 *Tdrd7-/-* and control lens microarray datasets, miRNA identification and miRNA-mRNA targets. **(B)** Comparisons of normalized expression intensities for three biological replicates for *Tdrd7-/-* and control using PCA analysis plots and **(C)** boxplot analysis. **(D)** Bar plot shows total number of miRNAs expressed at P4. Distribution of expression intensity for miRNAs is shown by *x*-axis while *y*-axis shows number of miRNAs expressed.

Tdrd7 expression in mouse embryonic lens fiber cells is highly enriched on both the transcript and protein levels (Figures 1A–C) and its germline knockout results in severe cataract defects by postnatal day (P) 22 (Figure 1D). While the mRNAs misexpressed in *Tdrd7-/-* mouse lens have been examined on the genomic level in previous studies (Lachke et al., 2011; Barnum et al., 2020), there is no information on the impact of Tdrd7 deficiency on lens miRNA expression. Therefore, we sought to examine *Tdrd7-/-* lens by global miRNA profiling (Figure 2A). Data on three biological replicates each for *Tdrd7-/-* and control lenses at postnatal day (P) 4 were generated on Affymetrix miRNA 3.0 microarrays. The stage P4 was selected for this analysis because it preceded cataract formation in *Tdrd7-/-* lenses (Figure 1D) and therefore was considered to yield information on the early alterations in miRNA expression, prior to the onset of lens defects, in turn reducing the possibility of detecting secondary miRNA changes. The microarray datasets generated were imported in R-statistical environment for systematic analysis of miRNAs expression in the *Tdrd7-/-* and control lens samples. The detailed bioinformatics pipeline is outlined in Figure 2A. The quality of miRNA microarray datasets was evaluated using Principal component analysis (PCA) (Figure 2B) and boxplot analysis (Figure 2C). Normalized miRNA expression intensity in PCA showed that *Tdrd7-/-* sample datasets (T1, T2, T3) were distinct from control datasets (C1, C2, C3) (Figure 2B). In P4 lenses, 697 mature miRNAs were detected with expression intensities ranging between 1.2 and 15592 (Supplementary Table 1). Further, the expression intensity distribution of these miRNAs showed that the vast majority (*n* = 503, 72.2%) are detected at expression intensity ≤ 5.0 (Figure 2D). Further, a majority [481 (95.6%)] of these 503 miRNAs were independently shown to be expressed in the mouse lens (Khan et al., 2015, 2016). The miRNAs found to be differentially expressed between *Tdrd7-/-* and control lens samples were identified in the 5-500 expression intensity range (Figure 2D).

### *Tdrd7-/-* Lens Exhibits Differentially Expressed miRNAs

**FIGURE 3:**
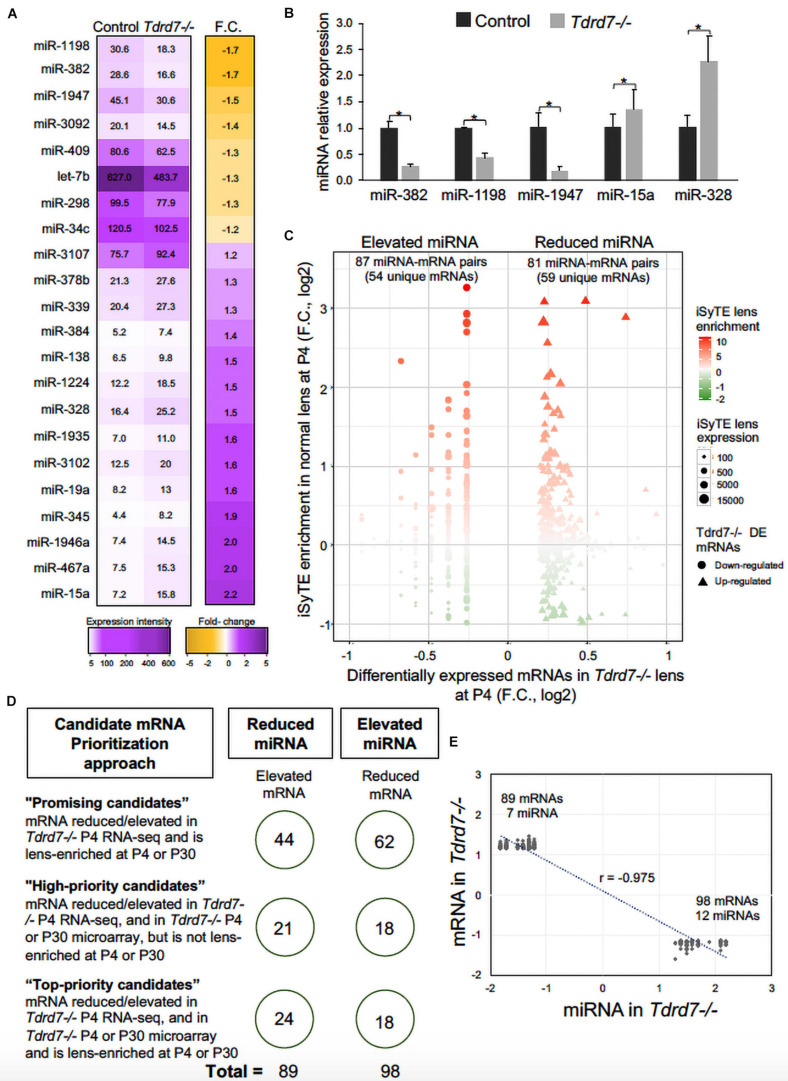
Mis-expression of miRNAs and their mRNA targets in *Tdrd7-/-* lens. **(A)** Heat-map represents comparison of miRNA expression in *Tdrd7-/-* and control lenses (left column) and mis-expression of twenty-two miRNAs (fold change (F.C.) ≥ ± 1.2 fold-change, *p* < 0.05) in *Tdrd7-/-* vs. control lenses (right column). **(B)** Relative miRNA expression in control and *Tdrd7-/-* lens. qPCR analysis independently validates significant reduction of miRNAs miR382, miR1198 and miR1947, and significant elevation of miRNAs miR15a and miR328. Error bars represents standard error of the mean. Asterisk represents *p*-value < 0.05. NS represents not significant *p*-values. **(C)** Lens-enriched expression of Tdrd7-downstream miRNA target mRNAs. mRNA targets of differentially expressed miRNAs in *Tdrd7-/-* lens (*x*-axis) are plotted against iSyTE enrichment scores at P4 (*y*-axis). Shape gradient represented as circles is for down-regulated mRNAs while triangles represent up-regulated mRNAs. Size gradient represents smaller to higher expression of mRNA. Red and green color gradients represent high and low lens-enrichment, respectively. Similar number of target mRNAs in the *Tdrd7-/-* lens reduced- and elevated-miRNA group exhibit lens enriched expression at stage P4. **(D)** Schematic shows candidate mRNA prioritization approach in the *Tdrd7-/-* lens reduced- and elevated-miRNA group. On the left are shown the rules that were used to prioritize candidate mRNAs in “Top-priority,” “High-priority,” and “Promising” candidate categories. On the right, shown in circles are the numbers of mRNA in the *Tdrd7-/-* lens reduced- and elevated-miRNA groups. **(E)** Pearson correlation analysis of miRNA and their target mRNAs identifies inverse correlation (r = -0.975) for the *Tdrd7-/-* lens reduced- and elevated-miRNA group.

We next compared miRNAs in *Tdrd7-/-* and control lens samples and identified 22 mis-expressed mature miRNAs at significant *p*-value (≤0.05) and absolute fold change (≥1.2) (Figure 3A). These included the following 8 miRNAs that were reduced in *Tdrd7-/-* lens: let-7b, miR-34c, miR-298, miR-382, miR-409, miR-1198, miR-1947, and miR-3092 (Table 1). The 14 miRNAs overexpressed in *Tdrd7-/-* lens were: miR-15a, miR-19a, miR-138, miR-328, miR-339, miR-345, miR-378b, miR-384, miR-467a, miR-1224, miR-1935, miR-1946a, miR-3102, and miR-3107.

**TABLE 1:** List of differentially expressed miRNAs in postnatal day 4 *Tdrd7-/-* lens.

miRNA	*Tdrd7 -/-*	Control	FC	*P*-value
mmu-mir-1198	18.3	30.6	−1.7	0.024
mmu-mir-382	16.6	28.6	−1.7	0.044
mmu-mir-1947	30.6	45.1	−1.5	0.048
mmu-mir-409	62.5	80.6	−1.4	0.019
mmu-mir-3092	14.5	20.1	−1.4	0.021
mmu-let-7b	483.7	627.0	−1.3	0.031
mmu-mir-298	77.9	99.5	−1.3	0.022
mmu-mir-34c	102.5	120.5	−1.2	0.044
mmu-mir-3107	92.4	75.7	1.2	0.049
mmu-mir-378b	27.6	21.3	1.3	0.046
mmu-mir-339	27.3	20.4	1.4	0.031
mmu-mir-384	7.4	5.2	1.4	0.018
mmu-mir-138	9.8	6.5	1.5	0.042
mmu-mir-1224	18.5	12.2	1.5	0.006
mmu-mir-1935	11.0	7.0	1.6	0.018
mmu-mir-328	25.2	16.4	1.6	0.024
mmu-mir-3102	20.0	12.5	1.6	0.008
mmu-mir-19a	13.0	8.2	1.7	0.049
mmu-mir-345	8.2	4.4	1.8	0.027
mmu-mir-1946a	14.5	7.4	1.9	0.035
mmu-mir-467a	15.3	7.5	2.1	0.010
mmu-mir-15a	15.8	7.2	2.2	0.012

### Prediction of Downstream mRNA Targets of Misexpressed miRNAs in *Tdrd7-/-* Lens

**FIGURE 4:**
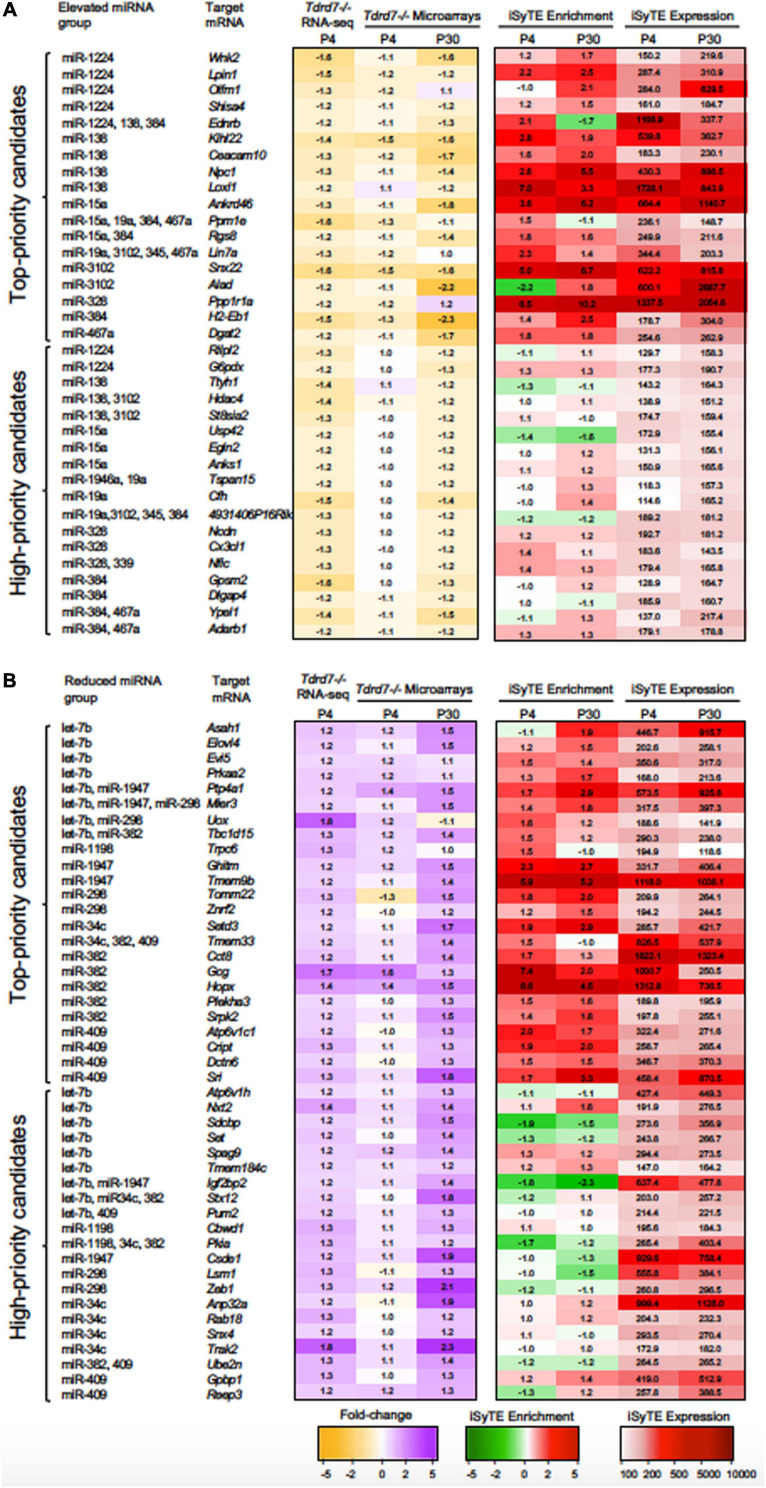
“Top-priority” and “High-priority” mRNA targets of Tdrd7-downstream miRNAs in the lens. Heat-maps representing expression data of “Top-priority” and “High-priority” mRNA targets of miRNAs identified in **(A)** “Elevated miRNA group” and **(B)** “Reduced miRNA group” in *Tdrd7-/-* lens. Differential expression in P4 (RNA-seq and microarray data) and P30 (microarray data) in *Tdrd7-/-* lens (left) and enriched expression and expression in normal lens as per iSyTE data at postnatal day (P) 4 and P30 (right). “iSyTE Enrichment” represents lens-enriched expression in fold change, while “iSyTE Expression” represents lens expression in fluorescence intensity units at P4 and P30. The mRNA targets are grouped based on the filtering criteria outlined in the text and in Figure 3C. Heatmap keys indicate the following: Yellow and purple color gradient represent low to high differential expression in *Tdrd7-/-* compared to control lens (in Fold-change) in RNA-seq data and microarray data. Green and red color gradients represent low to high lens-enrichment (in Fold-change) in iSyTE microarray data. Light-red and red gradients represent low to high lens-expression (in fluorescent intensity units) in iSyTE microarray data.

**FIGURE 5:**
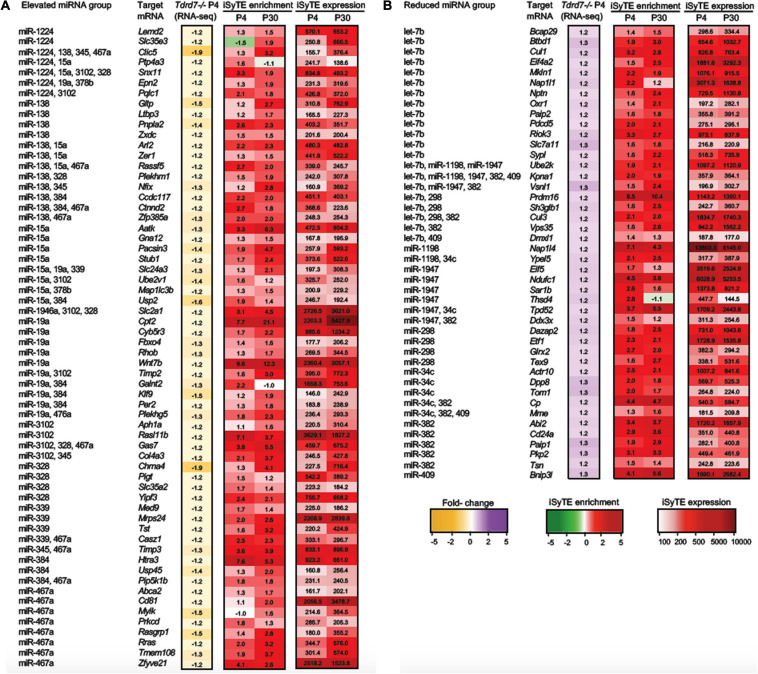
“Promising” mRNA targets of Tdrd7-downstream miRNAs in the lens. Heat-maps representing expression of “Promising” mRNA targets of miRNAs identified in **(A)** “Elevated miRNA group” and **(B)** “Reduced miRNA group” in *Tdrd7-/-* lens. The mRNAs identified as “Promising” targets in the “Elevated miRNA” group are identified as reduced in *Tdrd7-/-* P4 RNA-seq, and are lens-enriched at P4 and/or P30. In the “Reduced miRNA” group the “Promising” targets are identified as elevated in *Tdrd7-/-* P4 RNA-seq and are lens-enriched at P4 and/or P30. Heatmap keys indicate the following: Yellow and purple color gradient represent low to high differential expression in *Tdrd7-/-* lens compared to control lens (in Fold-change) in RNA-seq data. Green and red color gradients represent low to high lens-enrichment (in Fold-change) in iSyTE microarray data at P4 and P30. Light-red and red gradients represent low to high lens-expression (in fluorescent intensity units) in iSyTE microarray data at P4 and P30.

MicroRNAs control the cellular proteome by directly binding to their target mRNAs and channeling them to degradation or by inhibiting their translation into protein (Pasquinelli, 2012; Manning and Cooper, 2017; Hentze et al., 2018). In both cases, direct binding of miRNA to their target mRNA is a critical step. Therefore, to gain insight into the downstream impact of the differentially expressed miRNAs in the *Tdrd7-/-* lens transcriptome, we sought to identify miRNA-mRNA target binding pairs using the miRDB resource (Chen and Wang, 2020). This identified potential 11450 and 7142 mRNA targets for 12 elevated and 7 reduced miRNAs, respectively. The database did not contain information on mRNA targets for the differentially expressed miRNAs miR-3107, miR-1935 and miR-3092, and therefore these miRNAs were not included in further downstream analyses. Next, from the numerous potential target mRNAs, we sought to prioritize key candidates. Therefore, we first analyzed these target mRNAs in the context of genome-wide RNA-seq data on P4 *Tdrd7-/-* lenses (Barnum et al., 2020) and normal lens enriched-expression at P4 using the iSyTE database (Kakrana et al., 2018). At P4, RNA-seq showed *Tdrd7-/-* lens exhibited mis-expression of 1982 reduced and 1832 elevated mRNAs (Table 2). Further, among the 1982 mRNAs reduced in the *Tdrd7-/-* lens, 574 unique mRNAs (∼29%) were found to be direct targets of 12 miRNAs elevated in the *Tdrd7-/-* lens (hereafter referred to as miRNA-mRNA pairs). Similarly, among the 1832 elevated mRNAs in the *Tdrd7-/-* lens, 535 (∼29%) mRNAs were found to be direct targets of 7 miRNAs reduced in the *Tdrd7-/-* lens (Table 2). Thus, 1109 mRNAs collectively mis-expressed and inversely correlated with 19 miRNAs in *Tdrd7-/-* P4 lenses. Next, the inversely correlated miRNA-regulated mRNAs were tested for lens enriched expression at stage P4 lens using the iSyTE database. We found that the target mRNAs in the 87 miRNA-mRNA pairs (representing 54 unique mRNAs; the reduced number for mRNA targets is due to multiple miRNAs sharing target mRNAs) exhibited lens-enriched expression for miRNAs elevated in the *Tdrd7-/-* lens (Figure 3B). The target mRNAs in the 81 miRNA-mRNA pairs (representing 59 unique mRNAs) exhibited lens-enriched expression for miRNAs reduced in the *Tdrd7-/-* lens (Figure 3B). These data showed that similar proportions of elevated or reduced miRNA target mRNAs exhibited lens-enriched expression in normal development. We extended this analysis by applying a set of distinct filters and by including lens expression data at a later stage (*i.e.*, P30). This strategy applied differential expression data in *Tdrd7-/-* lens at both P4 and P30 and/or normal lens expression at P4 and P30 to identify miRNA-mRNA pairs as either “top-priority”, “high-priority” or “promising” candidates (Figure 3C). There were 154 miRNA-mRNA pairs (representing 98 unique mRNA targets) for elevated miRNAs and 123 (representing 89 unique mRNA targets) miRNA-mRNA pairs for reduced miRNA. For the elevated miRNAs, these include 18 top-priority candidates, 18 high-priority candidates and 62 promising candidates. For the reduced miRNAs, these include 24 top-priority candidates, 21 high-priority candidates and 44 promising candidates. Further, the inverse association of 154 elevated (98 unique target mRNAs reduced in *Tdrd7-/-* lens, also termed as belonging to “elevated miRNA group”) and 123 reduced miRNA-mRNA pairs (89 unique target mRNAs elevated in *Tdrd7-/-* lens, also termed as belonging to “reduced miRNA group”) was validated by performing Pearson correlation analysis. The correlation coefficient (r) for elevated and reduced miRNAs with their target mRNAs was -0.975 at a significant *p*-value of 0.00001 (Figure 3D). The “top-priority”, “high-priority” and “promising” mRNAs that are targets of elevated and reduced miRNAs – which are also identified among differentially expressed mRNAs in *Tdrd7-/-* lenses – are presented as heat map in the context of normal lens gene expression (Figures 4, 5).

**TABLE 2:** miRNA-mRNA pairs differentially expressed in Postnatal day 4 *Tdrd7-/-* lens.

miRNA group	miRNA in *Tdrd7-/-* (FC ± 1.2)	mRNA in *Tdrd7-/-* (FC ± 1.2)	miRNA-mRNA pairs in *Tdrd7-/-* P4 lens	miRNA-mRNA pairs enriched in iSyTE at P4	miRNA-mRNA pairs enriched in iSyTE at P30
Up	14	1982 (down)	915/1982 (46.16%) (representing 574/1982 (28.96%) unique mRNAs)	87 (9.50%) (representing 54 unique mRNAs)	128 (13.98%) (representing 71 unique mRNAs)
Down	8	1832 (up)	781/1832 (42.63%) (representing 535/1832 (29.20%) unique mRNAs)	81 (10.37%) (representing 59 unique mRNAs)	96 (12.29%) (representing 64 unique mRNAs)

We next examined the conservation of miRNA seed type in target mRNAs across vertebrates using the TargetScan database. In the elevated miRNA group, the following miRNA target mRNAs were conserved across vertebrates: miR-15a target mRNAs- Egln2 and Aatk; miR-19a target mRNAs- Epn2, Plekhg5 and Timp2; miR-138 target mRNA Nfix. While Egln2 and Plekhg5 have a 7mer conserved seed type, Aatk, Epn2, Timp2 and Nfix have an 8mer conserved seed type. In the reduced miRNA group, the following target mRNAs were conserved across vertebrates: let-7b target mRNAs Sh3glb1, Elovl4, Bcap29; and miR-34c target mRNA Tom1 were conserved at 7mer seed type, while miR-34c target mRNA Tpd52 was conserved at 8mer seed type.

### Functional Insights Into mRNA Targets of miRNAs in *Tdrd7-/-* Lens

**FIGURE 6:**
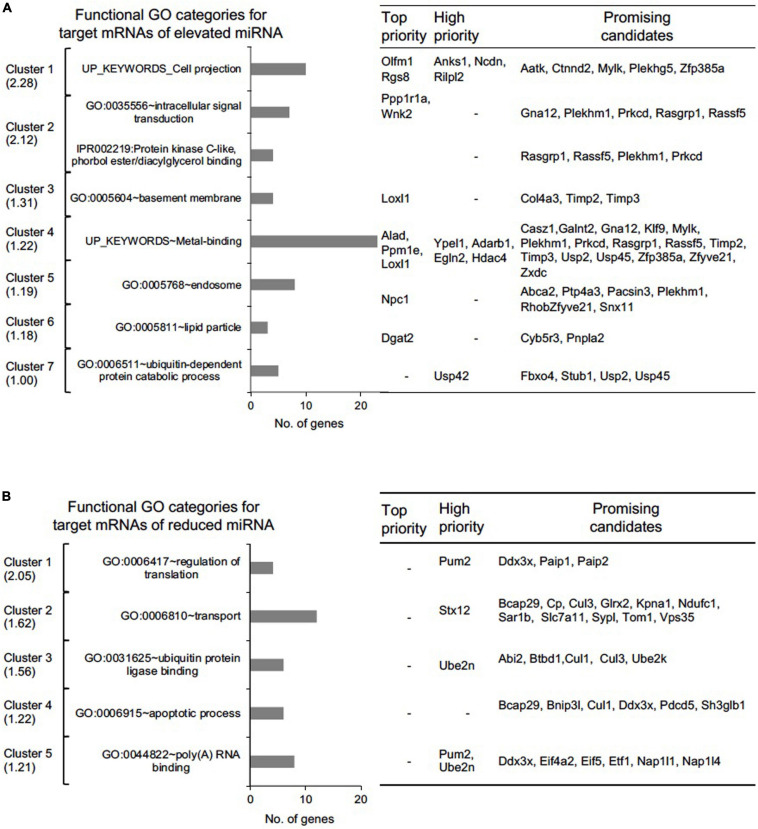
Functional gene ontology (GO) assessment of “Top-priority,” “High-priority,” and “Promising” mRNA targets of differentially expressed miRNAs in *Tdrd7-/-* lens. Functional GO analysis of **(A)** 154 miRNA-mRNA pairs representing 98 unique mRNA targets for elevated miRNAs and **(B)** 123 miRNA-mRNA pairs representing 89 unique mRNA targets for reduced miRNA using DAVID bioinformatics tool. Candidate mRNAs were grouped in GO clusters, which are presented from high to low cluster enrichment score (left panel). One or more relevant GO category in a cluster is presented as bar graph (middle panel). Number of candidate mRNAs in each cluster are grouped as top, high and promising candidates (right panel).

The prioritized candidates from the above analysis represent numerous miRNA-target mRNA genes whose functions are relevant to the observed lens defects in *Tdrd7-/-* mice. To systematically associate cellular function we imported these unique mRNAs (98 from elevated and 89 from reduced miRNA group, please see above) for analysis using the DAVID bioinformatics tool, and identified functional gene clusters (Figure 6 and Supplementary Tables 2, 3). Notably, several significantly enriched gene ontology (GO) categories (enrichment score > 1.0, *p* < 0.05) for the elevated and reduced miRNA groups are relevant to Tdrd7 function in the lens (Figures 6A,B). For example, for elevated miRNA group (*i.e.*, reduced mRNAs in *Tdrd7-/-* lens), GO:0042995 “cell projection” contained ten genes. These candidates were Rgs8, Olfm1 (top-priority), Rilpl2, Ncdn, Anks1 (high-priority) and Zfp385a, Plekhg5, Ctnnd2, Mylk, Aatk (promising category). Further, GO:0005604 “basement membrane” contained four genes including Loxl1 (top-priority), Col4a3, Timp2, Timp3 (promising category). This suggests Tdrd7 functions in downstream control of genes involved in cell-cell interaction or connectivity, in turn suggesting their potential impact on *Tdrd7-/-* cataract pathology, which is associated with vacuole-like gaps in fiber cells and posterior capsule rupture (Lachke et al., 2011; Tanaka et al., 2011; Barnum et al., 2020). Other interesting top clusters with GO categories for elevated miRNA group were GO:0035556 “intracellular signal transduction” (with promising candidates Gna12, Rasgrp1, Rassf5, Plekhm1, Prkcd), UP_keyword “metal binding” (with top-priority candidates Alad, Ppm1e, Loxl1, high- priority candidates Ypel1, Adarb1, Egln2, Hdac4), GO:0005811 “lipid particle” (with top-priority candidate Dgat2, and promising candidates Cyb5r3 and Pnpla2). On the other hand, the top clusters (enrichment score > 1.2, *p* < 0.05) in reduced miRNA group (*i.e.*, elevated mRNAs in *Tdrd7-/-* lens) were enriched in GO categories such as GO:0044822 “poly(A) RNA binding”, GO:0006915 “apoptotic process”, GO:0031625 “ubiquitin protein ligase binding”, GO:0006810 “transport” and GO:0006417 “regulation of translation” (Figure 6B). The upregulated genes (*i.e.*, reduced miRNA group) such as Pum2, Ddx3x are commonly found in the GO categories of poly(A) RNA binding and regulation of translation and Ddx3x is also present in the GO for apoptotic process. These GO categories defining the differentially expressed target mRNAs could help explain how misregulation of genes result in cataract. For example, these elevated targets could represent an attempt by lens cells to correct for the defects in post-transcriptional control resulting from Tdrd7-deficiency. Furthermore, elevated transcripts for genes associated with apoptotic process could help explain the lens defects such as large “gaps” (sometime referred to as “vacuoles”) in the fiber cell compartment of *Tdrd7-/-* mice.

### Tdrd7-Downstream miRNA-Based Coordinated Control in the Lens

Notably, miRNAs can function individually or in a coordinated manner to mediate regulation of their target mRNAs. A single miRNA can regulate multiple target mRNAs in a specific pathway or multiple miRNAs can converge on a single target mRNA to mediate its control. To gain insights into such coordinated control events potentially mediated by Tdrd7-downstream miRNAs in the lens, we examined coregulatory relationships among the “top-priority”, “high-priority” and “promising” mRNAs (as defined above). An individual miRNA can have multiple targets that are commonly identified in the same GO category. For example, the upregulated miRNA, miR-138, targets the mRNAs encoding Plekhm1 and Rassf5 that are commonly found in GO category “signal transduction”, while miR-345 targets Col4a3 and Timp3 mRNAs that are commonly found in the GO category “basal membrane”. On the other hand, we found single miRNA targeting mRNAs involved in varying cellular function. For example, miR-1224 targets eight mRNAs with diverse functions, namely, the enzymes G6pdx (dehydrogenase), Lpin1 (phosphohydrolase) and Wnk2 (kinase), an ER-localized protein with unknown function (Olfm1), a member of Shisha family (Shisa4), a lysosome morphology regulator (Rilpl2), a transmembrane protein (Lemd2) and a solute carrier family protein (Slc35e3). Similarly, miR-15a alone targets eight mRNAs such as Aatk (apoptosis associated tyrosine kinase), Ankrd46 and Anks1 (both associated with cytoskeletal regulations), Egln2 (oxygen homeostasis), Gna12 (signaling), Pacsin3 (protein kinase C involved in linking actin cytoskeleton with vesicle formation), Stub1 (ubiquitin ligase/co-chaperone) and Usp42 (de-ubiquitination). Other such miRNAs that alone target multiple mRNAs are miR-19a, miR-138, miR-328, miR-384, miR-3102 and miR-467a. Conversely, in the elevated miRNA group *(i.e.*, with reduced target mRNAs in *Tdrd7-/-* lens), 39 mRNAs were a common target for multiple miRNAs (Figures 4, 5). For example, both miR-384 and miR-15a have two targets in common, namely those encoding a protein phosphatase (Ppm1e) and a G-protein regulator (Rgs8) in the GO categories “cell projection” and “metal binding”, respectively. Further, six mRNAs with varying cellular functions such as Clic5 (involved in actin-based cytoskeletal structures), Ednrb (a receptor molecule), Lin7a (involved in maintaining cell membrane receptors and channels), Ppm1e (serine/threonine-protein phosphatases), Snx11 (a member of the sorting nexin family), 4931406P16Rik (unknown function) are common targets of different combinations of four miRNAs that are elevated in the *Tdrd7-/-* lens. Interestingly, for upregulated miRNAs, cohorts of mRNA identified in key GO categories were found to be targets of multiple miRNAs. For example, mRNAs involved in Ras signaling (Rasgrp1, Rasl11b, Rassf5, Rhob, Rras) were targets of multiple combinations of miRNAs (*e.g.*, miR-138, miR-15a, miR-19a, miR-3102 and miR-467a). Similarly, mRNAs encoding proteins in solute carrier family (Slc24a3, Slc2a1, Slc35a2, Slc35e3), metallopeptidases (Timp2, Timp3), proteins related to ubiquitin (Ube2v1, Usp2, Usp45) and zinc-finger proteins (Zfp385a, Zfyve21, Zxdc) were targets of multiple combinations of miRNAs.

In the reduced miRNA group *(i.e.*, with elevated target mRNAs in *Tdrd7-/-* lens), a similar trend in miRNA-mRNA connectivity was identified. For example, 23 mRNAs were a common target for multiple miRNAs (Figures 4, 5). Interestingly, the reduced miRNAs let-7b, miR-1198, miR-1947, miR-382 and miR-409 share a common mRNA target Kpna1[karyopherin (importin) alpha 1]. Further, combinations of multiple reduced miRNAs (*e.g.*, let-7b, miR-34c, miR-382, miR-1198, miR-1947, miR-298 and miR-409) commonly target the following mRNAs: Cul3 (polyubiquitination), Pkia (protein kinase inhibitor alpha), Stx12 (syntaxin), Ube2k (ubiquitin conjugating enzyme) and Vsnl1 (calcium sensor), which are found to be reduced in the *Tdrd7-/-* lens. Conversely, a single reduced miRNA, let-7b, has multiple mRNA targets, namely Cul1, Bcap29, Pdcd5 and Sh3glb1 that are commonly categorized in GO term ‘apoptotic process’ and are up-regulated in *Tdrd7-/-* lens. This data suggest that upregulated miRNAs have common targets that are involved in similar cellular pathways and are misregulated in *Tdrd7-/-* lens. In the reduced miRNA group, mRNAs encoding translation initiation factors (Eif4a2, Eif5, Etf1), nucleosome assembly proteins (Nap1l1, Nap1l4), polyadenylate binding proteins (Paip1, Paip2) were regulated by one or more different combination of miRNAs (let7b, miR1947, miR-1198, miR298 and miR-382), revealing miRNA-mRNA connectivity and their promising function in *Tdrd7-/-* lens (Figure 6B). Together, these analyses uncover the complexity of the Tdrd7-downstream miRNA-based control in the lens.

### Derivation of Common Regulatory Networks Between Tdrd7-Downstream miRNA-mRNAs and Other Key Lens Regulators

**FIGURE 7:**
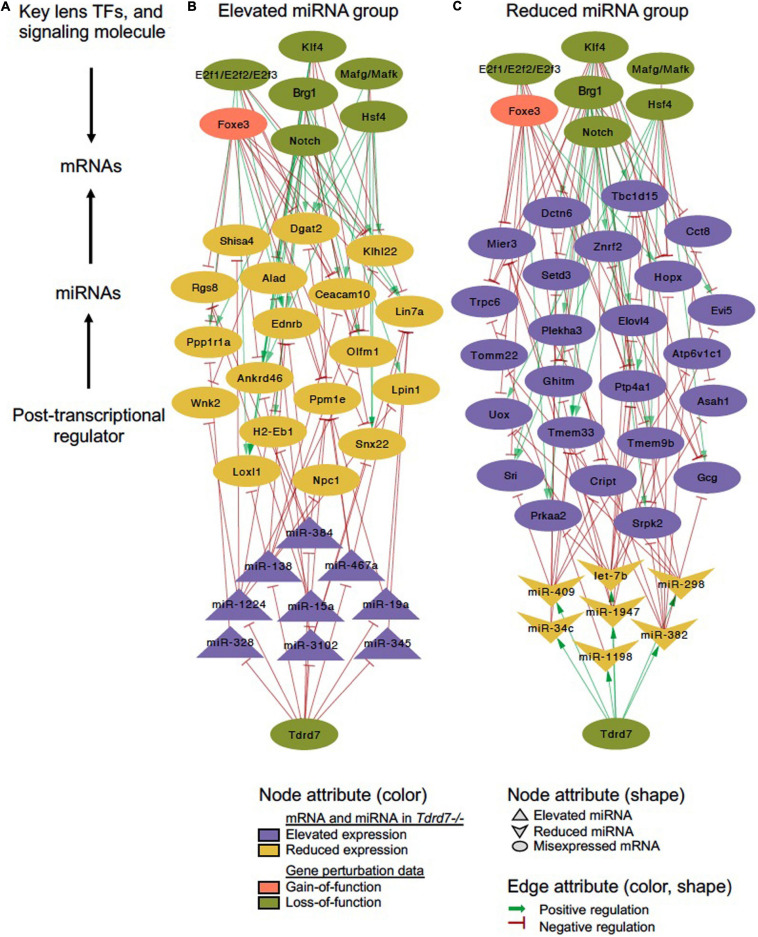
*Tdrd7* downstream global miRNA network and connectivity with key regulators in the lens. Global network analysis of misexpressed miRNA and mRNA targets in *Tdrd7-/-* lens reveals their connectivity with known lens regulators, namely the transcription factors, Brg1, E2f1/E2f2/E2f3, Foxe3, Hsf4, Klf4, Mafg/Mafk, and the signaling molecule, Notch. **(A)** schematic of global network connectivity of key factors in the *Tdrd7-/-* lens. **(B,C)** A regulatory network based on elevated and reduced miRNAs in *Tdrd7-/-* lens and their connectivity with target mRNAs, majority of which are also downstream of several key lens transcription/signaling factors. The edges color/shape represent positive regulation (dark green, arrow) and negative regulation (red, inhibitory sign) between miRNAs or transcription/signaling factors and mRNA targets. The transcription/signaling factor perturbation data in various mouse models is obtained from independent studies using gain-of-function (pink) or loss-of-function (pale green) approaches (details in the Methods section). Node shapes represents miRNA (elevated/reduced), mRNA and transcription/signaling factors.

**FIGURE 8:**
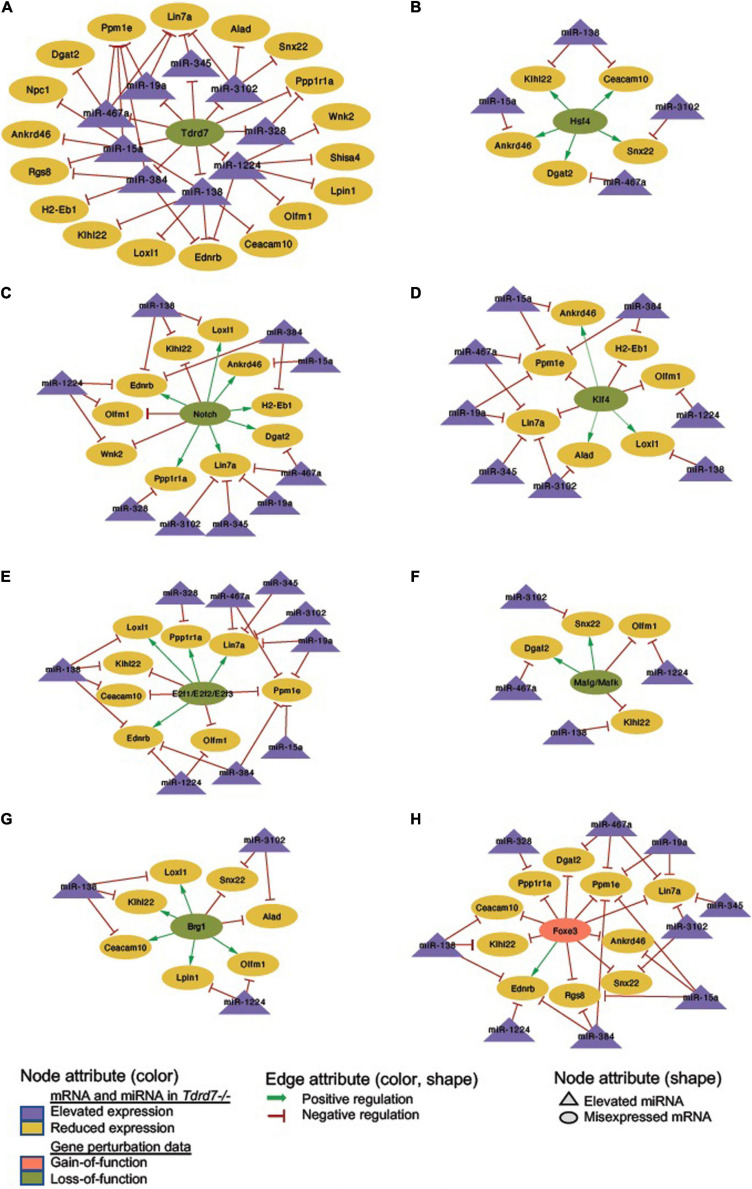
Regulatory networks for Tdrd7-downstream negatively regulated miRNAs and their mRNA targets and the relationship of these mRNA target with key lens regulators. **(A)** Network depicting Tdrd7 downstream negatively regulated miRNAs and the relationship with their predicted target mRNAs based on interpretation of *Tdrd7-/-* lens transcriptomics data. Deletion of Tdrd7 (pale green node) results in elevation of downstream miRNAs and therefore the relationship between Tdrd7 and these miRNA in normal lens is shown by red color inhibitory edge. In the *Tdrd7-/-* lens, this elevation of miRNAs results in repression of their target mRNAs, which are found to be reduced. Therefore, the relationship between the miRNAs and these target mRNAs in normal lens is indicated by red color inhibitory edge. Similarly derived networks based on lens microarray data on loss-of-function conditions of **(B)** Hsf4, **(C)** Notch, **(D)** Klf4, **(E)** E2f1/E2f2/E2f3, **(F)** Mafg/Mafk, **(G)** Brg1, and gain-of-function (in fiber cells) of **(H)** Foxe3 shows the regulatory relationship between these lens regulators and the Tdrd7-downstream negatively regulated miRNAs in the lens. The key provides information on the Edges, Nodes and gene expression conditions and directionality.

**FIGURE 9:**
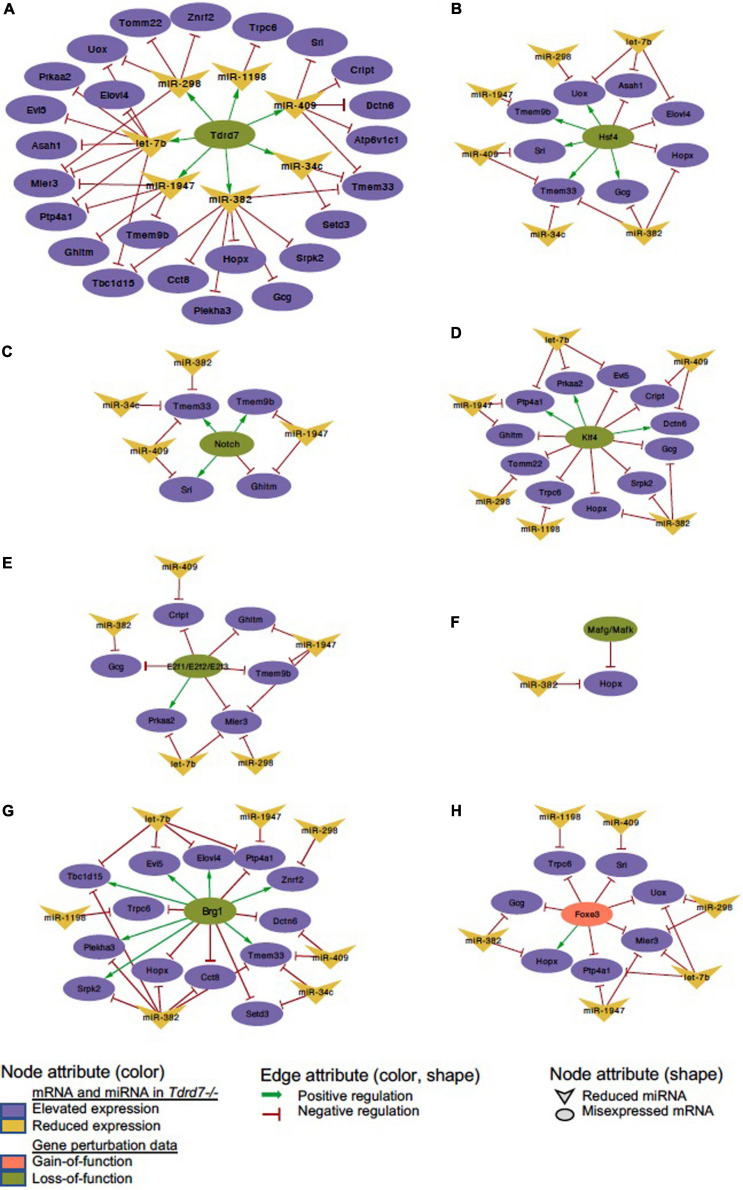
Regulatory network for Tdrd7-downstream positively regulated miRNAs and their mRNA targets and the relationship of these mRNA target with key lens regulators. **(A)** Network depicting Tdrd7 downstream positively regulated miRNAs and the relationship with their predicted target mRNAs based on interpretation of *Tdrd7-/-* lens transcriptomics data. Deletion of Tdrd7 (pale green node) results in reduction of downstream miRNAs, thus representing positive control by Tdrd7 in normal lens (therefore indicated by green arrow edge). This results in release of repression of the predicted target mRNAs of these miRNAs in the *Tdrd7-/-* lens (where these mRNA are found to be elevated) and thus the relationship between miRNA and these mRNAs in normal lens is indicated by red color inhibitory edge. Similarly derived networks based on lens microarray data on loss-of-function conditions of **(B)** Hsf4, **(C)** Notch, **(D)** Klf4, **(E)** E2f1/E2f2/E2f3, **(F)** Mafg/Mafk, **(G)** Brg1, and gain-of-function (in fiber cells) of **(H)** Foxe3 shows the regulatory relationship between these lens regulators and the Tdrd7-downstream positively regulated miRNAs in the lens. The key provides information on the Edges, Nodes and gene expression conditions and directionality.

We next sought to examine which downstream mRNAs are common between Tdrd7-regulated miRNA targets and those of other key regulators implicated in lens development and cataract. We focused on the top-priority Tdrd7-downstream mRNA targets for both elevated and reduced miRNA groups (*i.e.*, mRNA targets of miRNAs elevated or reduced in *Tdrd7-/-* lens; see above) and examined which of these were also misexpressed in different gene-specific perturbation (either gene-specific knockout, dominant negative or overexpression, representing loss-of-function or gain-of-function conditions) mouse models of lens defects/cataract that were subjected to meta-analysis in iSyTE (Kakrana et al., 2018). For key lens regulators, we focused on various transcription factors (*e.g.*, Brg1, E2f1/2/3, Foxe3, Hsf4, Klf4, Mafg, Mafk) and signaling pathways (*e.g.*, Notch) (Landgren et al., 2008; He et al., 2010; Saravanamuthu et al., 2012; Gupta et al., 2013; Agrawal et al., 2015; Anand et al., 2015). Based on this analysis, we derived a regulatory network which demonstrates how signaling, transcription and post-transcriptional regulatory pathways mediate combinatorial control over expression of key genes in the lens (Figure 7). To gain insight into the connectivity between Tdrd7-downstream miRNA-mRNA pairs and the above key lens defects/cataract-linked genes, we derived individual regulatory modules that effectively discern the node-edge relationship for individual gene perturbation conditions (Figures 8, 9). The regulatory network module for Tdrd7 shows its relationship with 18 downstream reduced mRNAs (Alad, Ankrd46, Ceacam10, Dgat2, Ednrb, H2-Eb1, Klhl22, Lin7a, Loxl1, Lpin1, Npc1, Olfm1, Ppm1e, Ppp1r1a, Rgs8, Shisa4, Snx22 and Wnk2) (Figure 8A). In normal lens, Tdrd7 negatively regulates 9 miRNAs in this network (miR-15a, miR-19a, miR-138, miR-328, miR-345, miR-384, miR-467a, miR-1224, miR-3102), which target these 18 mRNAs (Figure 8A). Majority of these mRNAs (16 out of 18; 89%) are also mis-expressed in seven other gene perturbation conditions (Figures 8B–H). Out of these 16, majority (81%) have one or more regulatory edge(s) in the same direction as in *Tdrd7-/-* lenses. Interestingly, Ankrd46, Ednrb, Dgat2 and Lox11 are all mis-expressed in multiple gene perturbation conditions and in the same direction as in *Tdrd7-/-* lenses. Next, we derived the regulatory network module for Tdrd7 displaying its relationship with 24 downstream elevated mRNAs (Figure 9A). In normal lens, Tdrd7 positively regulates 7 miRNAs in this network (let-7b, miR-34c, miR-298, miR-382, miR-409, miR-1198, miR-1947), which target these 24 mRNAs (Figure 9A). Almost all (23 out of 24) of these mRNAs are also mis-expressed in the seven other gene perturbation conditions (Figures 9B–H). Out of these 23, a majority (78%) have at least one regulatory edge in the same direction as in *Tdrd7-/-* lenses. Interestingly, Cript1, Gcg, Ghitm, Hopx, Mier3, Ptp4a1, Trpc6 are all mis-expressed in multiple gene perturbation conditions and in the same direction as in *Tdrd7-/-* lenses. Overall, these analyses suggest that many distinct regulatory pathways converge on common targets to mediate precise control over their expression in the lens. Finally, fibroblast growth factor signaling has been shown to control lens fiber cell differentiation (Padula et al., 2019) and Tdrd7 expression and function has been shown to be important in fiber cells as well (Lachke et al., 2011; Barnum et al., 2020). Previously, miRNA profiling has been performed on FGF2-induced rat lens explants (Wolf et al., 2013). To identify miRNA common to these pathways, we next compared Tdrd7-downstream miRNAs (present study) with the FGF2-induced lens explant miRNAs dataset (Wolf et al., 2013). In both studies, miRNAs miR-138, miR-328, miR-345 were commonly found to be significantly elevated, indicating that Fgf and Tdrd7 regulatory pathways potentially converge to mediate control over select downstream miRNAs in lens fiber cells.

### Microarray Profiling Identifies Highly Expressed miRNAs in Early Postnatal Lens

Finally, the miRNA profiling by microarray allows an opportunity to assemble a global catalog of the different miRNAs that are robustly expressed in the early postnatal mouse lens. Therefore, we next examined the highly expressed miRNAs (*n* = 31) defined as those having expression intensity ≥ 500 (*p* ≤ 0.05) in the control P4 lens (Table 3). This analysis identified 26 new highly expressed miRNAs in the lens while also validating the high expression of previously identified miRNAs (Supplementary Table 4). For example, the miRNAs miR-5105, miR-5109, miR-1298 and miR-378 were newly identified to be highly expressed in the lens. Further, this work offered independent support for the high expression of numerous miRNAs that were previously described in the lens. For example, the present study validates miRNA expression in the mouse lens in agreement with previous studies using microarrays, RNA-sequencing, and *in situ* hybridization (Supplementary Table 4) (Conte et al., 2010; Karali et al., 2010; Khan et al., 2016). Interestingly this analyses also identified several miRNAs (miR-184, miR-26a, miR-204, let-7b and let-7c) (Table 3) that were previously found to be misexpressed in human cataractous lenses, again offering independent support that these miRNAs are of significance to lens biology and cataract (Wu et al., 2012, 2017). Further, many FGF2-regulated miRNAs that were described in a previous study (Wolf et al., 2013) were also found to be highly expressed in the lens in the present study, offering independent support that miRNA function is important in lens development. Thus, this study identifies many miRNAs with high expression in the lens, which can be candidates for future investigations in lens development and cataract pathology.

**TABLE 3:** Highly expressed miRNAs in Postnatal day 4 mouse lens.

miRNA ID	miRNA expression intensity at P4
mir-184	15592.3
mir-709	8631.6
mir-31	5871.0
let-7e	3255.7
mir-26a	3129.2
mir-17	2697.1
mir-181a	2298.5
mir-181b	2227.2
mir-125a	2226.9
mir-99b	2181.6
mir-20a	1956.3
let-7c-1	1708.8
mir-24	1397.8
mir-125b	1339.0
mir-103	1280.4
mir-23b	1133.3
mir-23a	1088.0
mir-191	947.9
mir-93	939.8
mir-107	910.4
let-7a	889.8
mir-5105	752.1
mir-16	727.8
mir-5109	684.1
mir-92a	634.0
let-7b	627.0
mir-106a	601.0
mir-130a	594.7
mir-5126	590.7
let-7d	590.4
mir-204	515.4

## Conclusion

These findings suggest that Tdrd7-downstream miRNAs function to maintain optimal levels and specificity of the mRNA transcriptome in the lens, the misexpression of which may contribute to cataract pathology. This is in line with the notion that many small changes orchestrated by several miRNAs may contribute toward fine-tuning gene expression in a cell/tissue (Lim et al., 2005). Regulatory connections between many genes relevant to lens biology and pathology were identified in this study. For example, candidates associated with cell projection, such as Mylk, Olfm1, Plekhg5 and Rgs8, among others, were in the reduced mRNAs (elevated miRNAs) category in the *Tdrd7-/-* lens. Further, in “intracellular signal transduction” category, Rasgrp1 was identified among other candidates. This is interesting because in an independent study we recently found Rasgrp1 to be involved in the rescue of fiber cell defects in *Fgfr2:Pten* compound null lenses (Padula et al., 2019). In the GO category “basement membrane”, Col4a3, Loxl1, Timp2 and Timp3 were identified as reduced mRNA candidates, which is relevant to the lens capsular defects observed in *Tdrd7*-defcient lenses, especially because *COL4A3* (Collagen Type IV Alpha-3) and LOXL1 (lysyl oxidase-like 1) mutations are associated with cataract and glaucoma in humans (Thorleifsson et al., 2007; Uzak et al., 2013). Further, this analysis also identified – among the promising candidates – the transcription factor *CASZ1*, which was previously predicted by iSyTE as potentially important in lens (Kakrana et al., 2018) and recently found in a genome-wide association study (GWAS) to be associated with cataract in humans (Choquet et al., 2020). Finally, several genes in the GO category “apoptotic process” were found to be elevated (reduced miRNAs) in the *Tdrd7-/-* lens, offering an explanation for the large vacuole-like gap defects observed in the fiber cells of Tdrd7-defecient cataractous lenses. To test individual or combinatorial contributions in the lens of the cohort of miRNAs described here, simultaneous gain/loss of function experiments involving these miRNAs will have be to performed in the future. Also, the present study does not assess the impact of these miRNAs on the level of proteins in the lens. This can be addressed in future studies determining the proteome profile of *Tdrd7-/-* lens at a stage prior to the onset of lens defects. It will also be interesting to examine the nature of control (whether direct or indirect) that is mediated by Tdrd7 over miRNAs in the lens. In sum, the data presented here indicate that Tdrd7 coordinates distinct downstream regulatory events–either through miRNA-mRNA interactions or through protein-mRNA interactions–to mediate post-transcriptional gene expression control in lens development, misregulation of which causes lens defects and congenital cataract.

## Data Availability Statement

The miRNA microarray datasets generated for this study can be found in the Gene Expression Omnibus database under accession number GSE157061.

## Ethics Statement

The animal study was reviewed and approved by University of Delaware Institutional Animal Care and Use Committee (IACUC).

## Author Contributions

DA, SA, CB, SS, SC, and SL contributed to the generation of the data. DA, SA, and SL analyzed the data. DA and SL wrote the manuscript. All the authors contributed to the revision of the manuscript.

## Conflict of Interest

The authors declare that the research was conducted in the absence of any commercial or financial relationships that could be construed as a potential conflict of interest.
